# IRIDIA-AF, a large paroxysmal atrial fibrillation long-term electrocardiogram monitoring database

**DOI:** 10.1038/s41597-023-02621-1

**Published:** 2023-10-18

**Authors:** Cédric Gilon, Jean-Marie Grégoire, Marianne Mathieu, Stéphane Carlier, Hugues Bersini

**Affiliations:** 1 IRIDIA, Université libre de Bruxelles, Brussels, Belgium; 2 Cardiology Department, Université de Mons, Mons, Belgium

**Keywords:** Atrial fibrillation, Databases, Machine learning, Databases

## Abstract

Atrial fibrillation (AF) is the most common sustained heart arrhythmia in adults. Holter monitoring, a long-term 2-lead electrocardiogram (ECG), is a key tool available to cardiologists for AF diagnosis. Machine learning (ML) and deep learning (DL) models have shown great capacity to automatically detect AF in ECG and their use as medical decision support tool is growing. Training these models rely on a few open and annotated databases. We present a new Holter monitoring database from patients with paroxysmal AF with 167 records from 152 patients, acquired from an outpatient cardiology clinic from 2006 to 2017 in Belgium. AF episodes were manually annotated and reviewed by an expert cardiologist and a specialist cardiac nurse. Records last from 19 hours up to 95 hours, divided into 24-hour files. In total, it represents 24 million seconds of annotated Holter monitoring, sampled at 200 Hz. This dataset aims at expanding the available options for researchers and offers a valuable resource for advancing ML and DL use in the field of cardiac arrhythmia diagnosis.

## Background & Summary

Cardiovascular diseases are one of the leading causes of death globally. To better treat them and help patients, research is necessary to have a deeper understanding of their various manifestations. In this work, we focus on atrial fibrillation (AF), the most common sustained heart arrhythmia in adults. The lifetime risk of AF is estimated 2% to 4% of the adult worldwide population^1,2^. The prevalence of the disease increases significantly with age, and for patients over 80, it is estimated around 20% of the population. In addition, due to the growing lifespan of the population and the intensified efforts to detect previously undiagnosed cases, projections indicate a twofold increase in AF prevalence in the coming years. Although the disease can be asymptomatic and can be considered as benign, patients with AF have a fivefold increased risk of stroke^3^ and a twofold increased risk of mortality^4^.

Electrocardiogram (ECG) is the primary non-invasive diagnostic tool available to cardiologists for detecting signs of AF. However, due to the paroxysmal nature of the disease in its early state, i.e. the disease starts and stops without known warning signs, the standard 10-second 12-lead ECG is not always able to capture AF episodes. Indeed, the patient can be in normal sinus rhythm during the brief recording window. Longer term monitoring methods, such as Holter monitoring, are used to overcome this duration limitation and have emerged as important tools in the diagnosis of AF. Holter recordings involve a 1-lead or 2-lead long-term monitoring of the patient’s cardiac activity, which typically lasts 24 hours but can be extended up to a week. By providing an extended view of the heart activity, Holter recordings offer the opportunity to detect and analyze paroxysmal AF episodes effectively.

Machine learning (ML) and deep learning (DL) advance in the last years had a consequent impact on the medical decision support field. DL models are able to detect arrhythmia with the same accuracy as cardiologists^5^ and to identify the signature of AF during sinus rhythm records a month before the first signs of AF^6^. These technological advancements could help to leverage short-term ECG and long-term Holter record data to diagnose and manage AF in the general population. However, the limited quantity of large-scale publicly available databases with annotations for the training and validation of DL models is a major obstacle for the research and the development of new medical tools. The most commonly used database for the development of ML model is the MIT-BIH arrhythmia database^7^ available on Physionet^8^. This dataset is a great resource, but the number of records (n = 47) and the record duration of 30 minutes limit the use of this model for the development of larger DL models.

In this work, we present IRIDIA-AF, a new large publicly available paroxysmal AF Holter monitoring database. The main objective of this database is to expand the available options for the development of ML and DL models for the detection of paroxysmal AF. The comparison of the IRIDIA-AF database and other publicly available database with AF diagnosis is presented in Tables 1, 2. Other databases, such as the PTB-XL^9^ or the AF classification challenge 2017 database^10^ propose a larger number of patients and number of heart disease diagnosis. IRIDIA-AF database proposes a larger record duration when compared to other publicly available databases with AF. The total duration of all records in the database represents more than 24 million seconds of records in total, which represent 278 days or 6690 hours of Holter recordings. In total, 388 AF episodes were recorded, with a total duration of 5 million seconds, which represent 67 days or 1609 hours. It corresponds to 24% of the total duration of the dataset. In addition, thanks to the length of the records, this database can also be used for other AF related tasks, such as short-term AF onset forecast^11^. Other databases, as PTB-XL, cannot be used for AF onset short-term forecast as the records are 10-second long and does not include the minutes before AF onsets.

**Table 1:** Comparison of publicly available ECG arrhythmia database and IRIDIA-AF.

Name	# patients	# records	# leads	record duration (seconds)		# classes
				min	max	
MIT-BIH Arrhythmia^7^	47	48	2	1800	1800	2
AF classification challenge 2017^10^	8528	8528	1	9	61	4
SPH dataset^13^	24 666	25 770	12	10	60	59
CU-SPH dataset^19^	10 646	10 646	12	10	10	11
PTB-XL^9^	18 885	21 837	12	10	10	71
IRIDIA-AF (this work)	152	167	2	71 408	345 596	2

**Table 2:** Comparison of available samples in publicly available ECG arrhythmia database.

Name	Total duration (seconds)	Sampling rate (Hz)	Total samples
MIT-BIH Arrhythmia^7^	86 400	360	31 104 000
AF classification challenge 2017^10^	277 138	300	83 141 400
SPH dataset^13^	281 109	500	140 554 500
CU-SPH dataset^19^	106 460	500	53 230 000
PTB-XL 100 Hz^9^	218 370	100	21 837 000
PTB-XL 500 Hz^9^	218 370	500	109 185 000
IRIDIA-AF (this work)	24 085 688	200	4 817 137 600

## Methods

This retrospective study was approved by the institutional ethics committee Erasme-ULB (P2017/413). The request for exemption from consent has been granted by the committee, due to the unrealistic feasibility of obtaining consent given the large number of involved cases and the high probability of being unable to reach numerous patients, and the publication of the anonymous data was allowed. The raw ECG signal data was recorded using Microport Spiderview Holter recorder. The data acquisition phases started in January 2006 and ended in August 2017, when the Ethics Committee’s application form was submitted. From the 10803 records, a total of 167 records from 152 patients were selected. The recording frequency of the device is 200 Hz, with a precision of 10 µV. Two leads were recorded: lead I and lead II. The medical analysis and annotation were done using Microport Syneview (version 3.30a).

The selection of records was carried as follows:

1. The Holter record database from Dr Jean-Marie Gregoire outpatient clinic, containing a total of 10803 records, was reviewed and searched by an experienced specialist cardiac nurse. Holter records from patients with Cardiac Implantable Electronic Device (CIED) were rejected. Holter with persistent or permanent AF, or other cardiac diseases were rejected. Holter presenting signs of AF were selected and put aside.
2. The selected records were reviewed by an experienced cardiologist to validate the diagnosis. Records with insufficient quality and excessive noise were rejected.
3. All the records passing the two previous validations were annotated. The annotation consists of searching and determining the precise beginning and end of each AF crisis in each record. The start of the AF crisis corresponds to the first beat in AF, as shown in Fig. 1. The annotation is positioned on the QRS complex of this first AF beat. The end of the AF crisis corresponds to the first beat in normal sinus rhythm (NSR) after the crisis. The annotation is also positioned on the QRS complex of this first NSR beat. In case of doubt about one event, a second opinion was asked to validate the annotation.

**Fig. 1:**
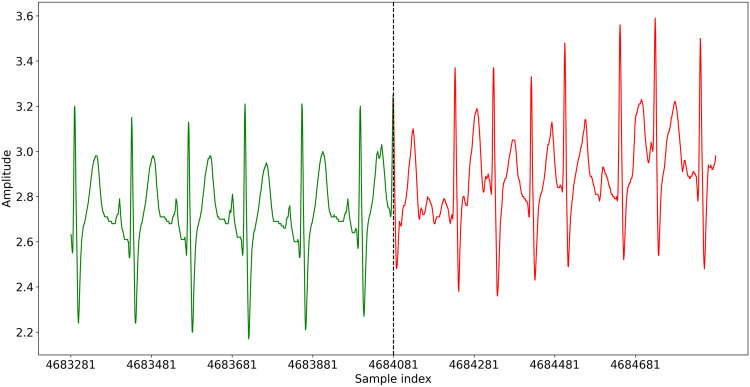
AF onset for the first AF crisis in ECG record record_026.
4. The record was then exported from Microport proprietary format to ISHNE format^12^ and stored along the annotations. The RR intervals resulting of the automatic QRS annotation by Microport Syneview software are exported in a second file.
5. The labels were checked by a technical expert to ensure the alignment with the waveform data available in the exported file. Because the recording frequency is 200 Hz and the annotations were accurate down to the second, the sample index that corresponds to the annotated time may not precisely align with the selected QRS complex. If a difference was found, the label was manually corrected to correspond precisely to the QRS complex index chosen by the annotators. An example of label and label correction is presented in Fig. 2.

**Fig. 2:**
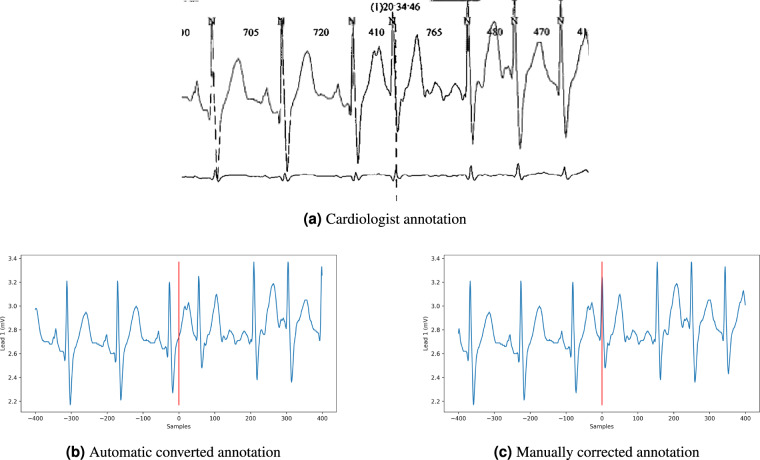
Example of annotation correction in record_026. The first annotation (**a**) is made by the cardiologist. The converted annotation (**b**) is slightly different because of the conversion from time to index. Therefore, it needs a manual correction (**c**) to precisely correspond to the chosen QRS complex.
6. For some records, the recording was not stopped just after the electrodes were removed from the patient skin. The end of each record was visually inspected to determine if *end of record* noise is present. An example of such *end of record* noise is presented in Fig. 3, where most of the record is noise. If *end of record* noise is present, the record was trimmed to only contain the interesting data. The RR files were automatically reworked to correspond to the new length of the file.

**Fig. 3:**
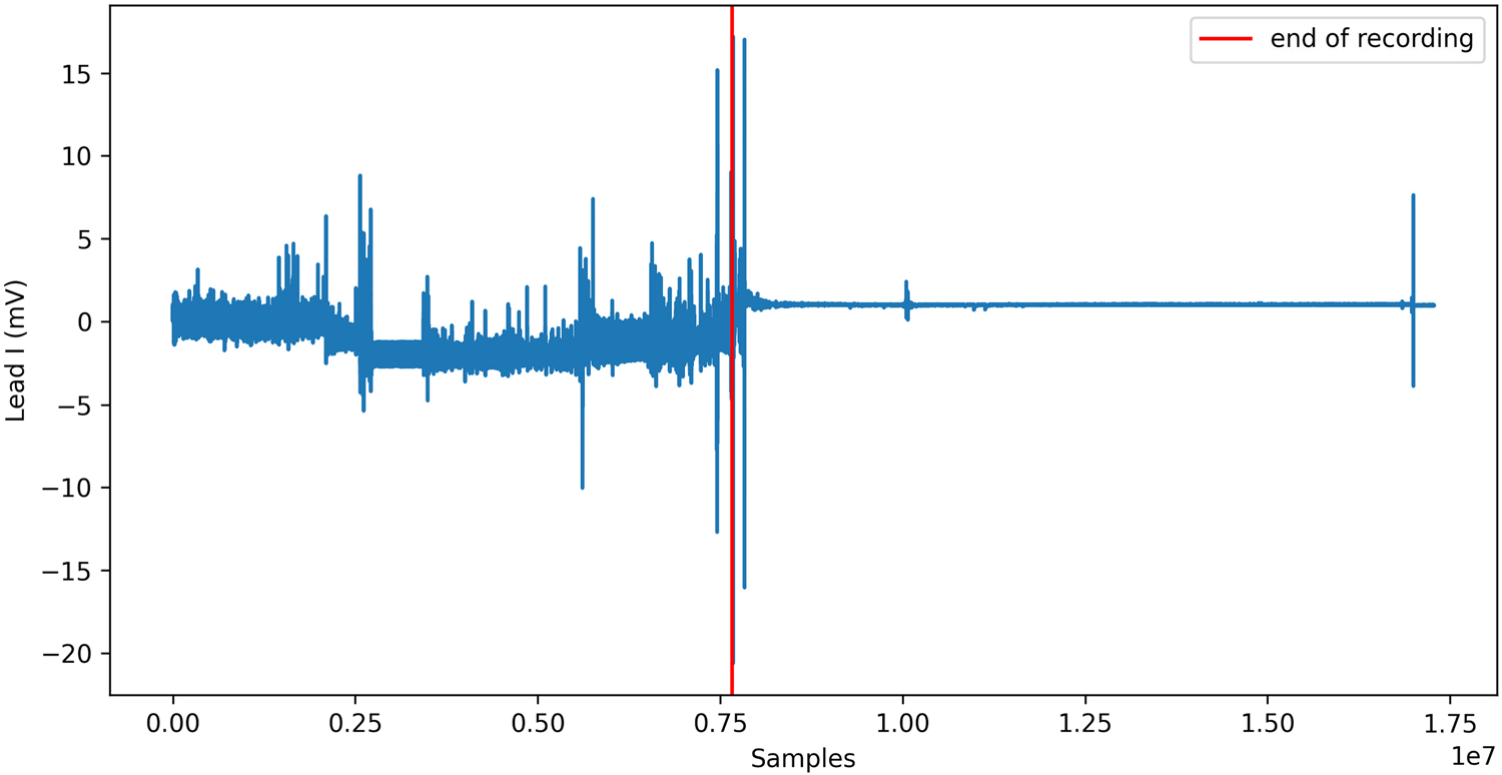
ECG record record_142 with noisy end after electrode removal.
7. The waveform files and RR files were exported from ISHNE format to HDF5 format. The metadata files were double-checked with annotations.

The quality of the waveform file has been left as produced by Microport software, to correspond to real-life records. Records with a high level of noise were discarded during the selection phase. The sampling frequency of the records was not altered and kept at 200 Hz. The unique patient identifier and unique record identifier were generated randomly. Patients can have multiple Holter records. Therefore, the records are associated to the same patient identifier. Each record acquisition date was shifted by a random offset for each patient, as proposed by previous ECG databases^9,13^. If there are multiple records for a patient, the chronological order of the records is conserved. We converted the birthdate of each patient, to its age at the time of the record.

## Data Records

**Fig. 4:**
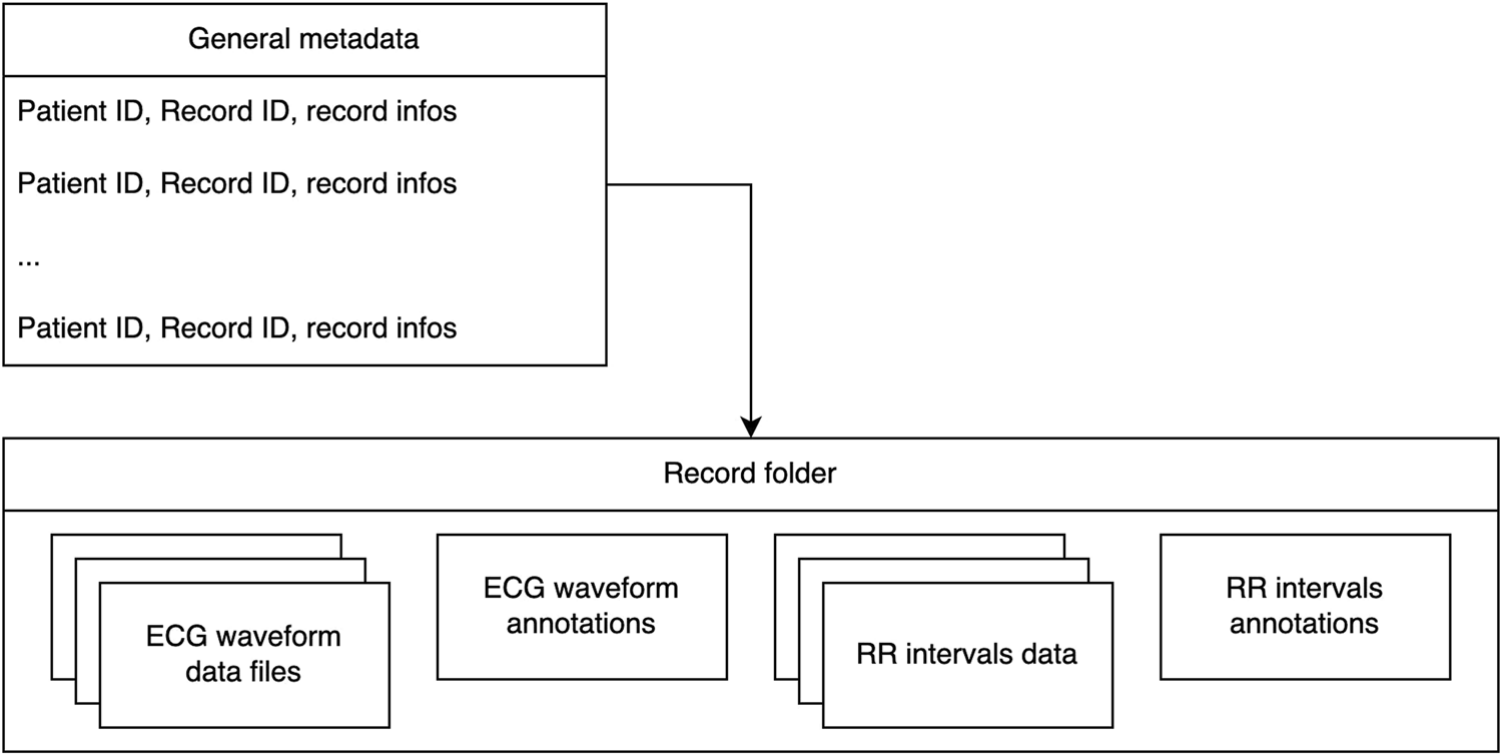
Files composition in the IRIDIA-AF database.

The database^14^ is available on Zenodo (https://zenodo.org/record/8405941). The IRIDIA-AF database is composed of a general metadata file and 167 folders, one for each record in the database. Each record folder includes the ECG waveform from the Holter record and the associated annotations. It also contains the RR intervals and associated annotations. This section describes the composition of the data repository. The composition is graphically described in Fig. 4.

### General metadata

**Fig. 5:**
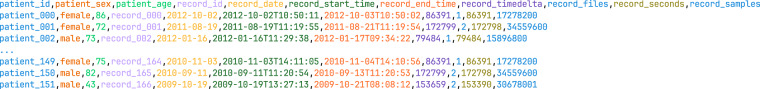
Content of the first and last lines in the general metadata file.

**Fig. 6:**
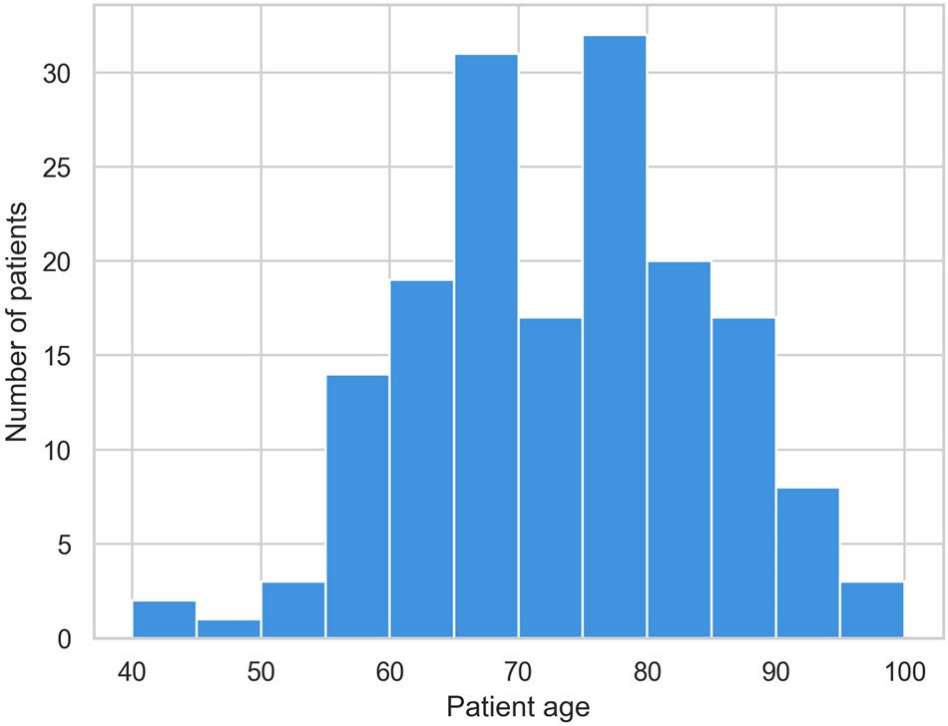
Distribution of patient age.

**Fig. 7:**
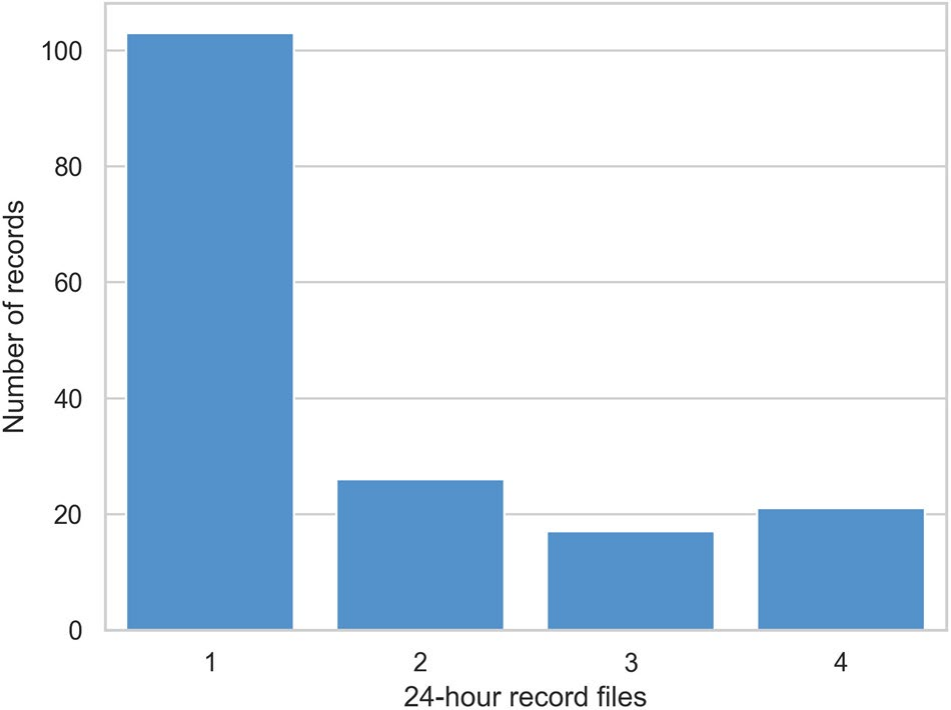
Distribution of record days (continuous period of 24 hours) per record.

**Fig. 8:**
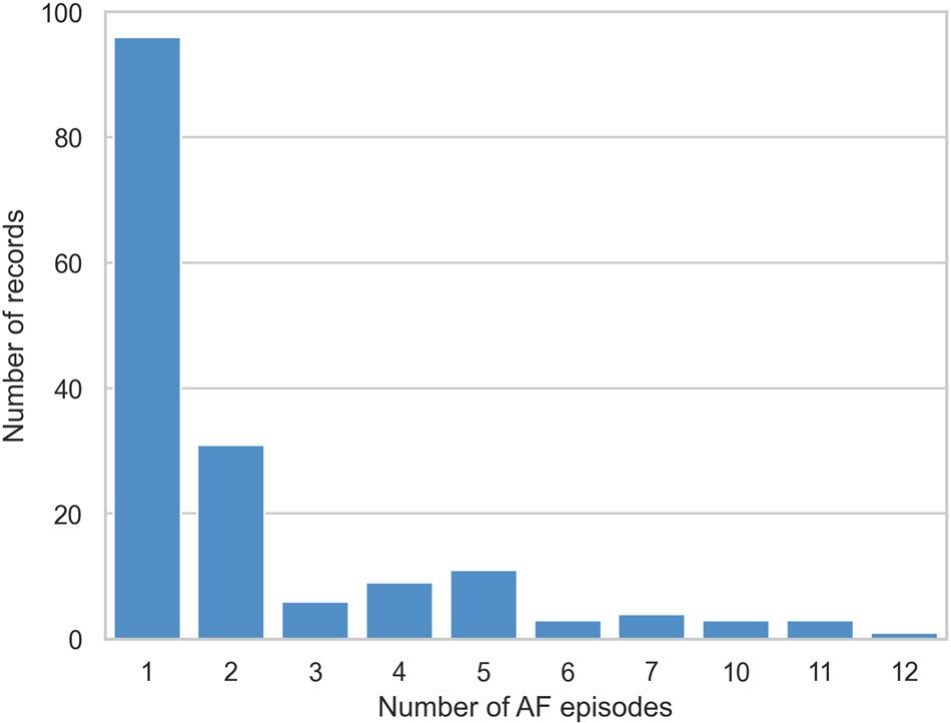
Distribution of the number of AF episodes per record.

We provide the general metadata about the record in a single table, contains in a *csv* format file as shown in Fig. 5. The file contains multiple columns with information about the patient and the record. The first columns contain information about the patient:

1. patient_id: the identifier of the patient;
2. patient_sex: the sex of the patient, i.e. male or female;
3. patient_age: the age of the patient at the day of the record, or the first day of record if there are multiple record days.
  The following columns contain general information about the record itself:
4. record_id: the identifier of the record;
5. record_date: the (shifted) date of the record;
6. record_start_time: the start time of the record in ISO 8601 format;
7. record_end_time: the initial end time of the record in ISO 8601 format;
8. record_timedelta: the time delta in seconds between the start and the end of the record.
  Finally, the following columns contain information about the files:
9. record_files: the number of ECG files for the record;
10. record_seconds: the real number of seconds in the record, i.e. this can differ from the record_timedelta due to the correction of the end of the record if noise was present;
11. record_samples: the real number of samples in all ECG files after end-of-file correction;

The age range is distributed between 41 and 99 years, with a mean age of 72 ± 11 years. The distribution is presented in Fig. 6. 53.2% are male and 46.7% are female. Mean CHADVASC score is 3.16 and range from 1 to 9. Holter are split into 24 hours record and most of the records (n = 103) have only one day of record, as shown in Fig. 7. In total, 388 AF episodes were recorded. Most of the records have only one (n = 96) or two (n = 31) AF episodes, but some records have up to 12 episodes, as show in Fig. 8.

### ECG waveform data

**Fig. 9:**
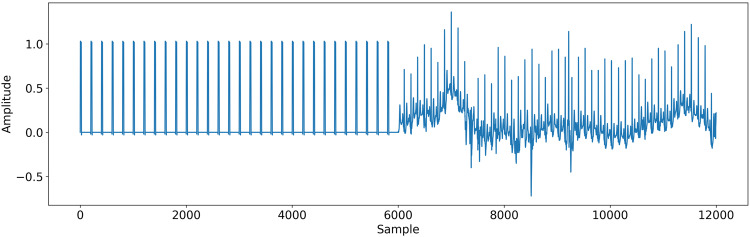
Calibration phase over the first 30 seconds of the ECG record record_077.

The ECG waveform data is stored in HDF5 format, in the form of an array of shape *L* × 2, where 2 correspond to the two leads (lead I and lead II) and *L* correspond to the number of records points, i.e. number of seconds × sampling frequency (200 Hz). This format is designed for data storage and supported by a wide variety of programming language. In addition, the compression level helped to reduce the dataset size without losing information quality and the data can be loaded in slices rather than having to load the whole file in memory. Each record is split in a multiple 24-hour part. Each part is stored in a separate HDF5 record associated with the record identifier and an identifier, e.g. record_000_ecg_00.h5 for the first 24-hour of record and record_000_ecg_01.h5 for the second 24-hour. The number of available ECG files is given in the general metadata file, stored in the record_n_files value. It should be noted that the first 30 seconds of record, i.e. from index 0 to index 6000, correspond to the calibration phase of the recording device, as shown in Fig. 9.

### ECG waveform annotations

For each record, one ECG waveform metadata file contains the annotations about each AF crisis with one AF onset, i.e. transition from NSR to AF, and one AF termination, i.e. transition from AF to NSR. Each line contains information about one crisis with the following information:

1. start_datetime: the day and time of the AF onset, in ISO 8601 format;
2. start_file_index: the number of the file in which the AF start;
3. start_qrs_index: the index of the QRS complex where the AF start, i.e. the first beat in AF;
4. end_datetime: the day and time of the AF termination in ISO 8601 format;
5. end_file_index: the number of the file in which the AF ends;
6. end_qrs_index: the index of the QRS complex of AF termination, i.e. the first NSR beat after the AF termination;
7. af_duration: the duration of the AF crisis in seconds;
8. nsr_before_duration: the duration of NSR before the AF onset, i.e. the time between this AF crisis and the previous AF crisis or the start of the record.

**Fig. 10:**
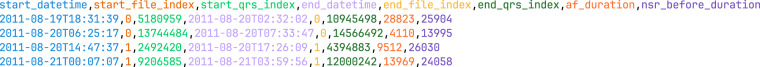
Content of ECG annotations file of record record_001.

An example is presented in Fig. 10. We chose to use the start and end keywords to represent AF onset and AF termination to make it as easy as possible to understand the file content. Records are split in 24-hour files and therefore, an AF event can be starting on the calendar date *d* and end in calendar day *d* + 1 and still be in the same 24-hour record file. AF can also extend over several days of recordings, e.g. an AF crisis can start in record 0 and end in record 1.

### RR intervals data

The RR intervals data file contains RR intervals derived from the automatic QRS annotations by Microport Syneview. The RR intervals are represented in milliseconds. The data is stored in HDF5 format, in the form of an array of length *L*, where *L* correspond to the number of RR intervals. As for the ECG, the first 30 second of RR intervals correspond to the calibration phase. Therefore, the first 30 RR intervals are equal to 1000 ms. It should be noted that this number may vary slightly from one file to another, as the Microport automatic annotation does not always produce similar analyses for this phase. As for the ECG waveform data, each record day is stored in a separate record.

### RR intervals annotations

**Fig. 11:**
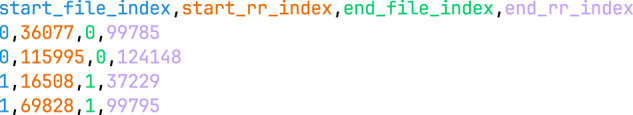
Content of the RR intervals annotations file of record record_001.

The RR intervals metadata files contain the information about AF crisis correspondence with automatic QRS detection. The data is presented in a *csv* file, containing one line for each AF crisis in the record, as shown in Fig. 11. The information are the following:

1. start_file_index: the index of the record with the AF onset;
2. start_rr_index: the index in the corresponding file where the AF start, i.e. the RR intervals with one beat in NSR and the following beat in AF;
3. end_file_index: the index of the record with the AF termination;
4. end_rr_index: the index in the corresponding file where the AF ends, i.e. the RR intervals with one beat in AF and one beat in NSR.

## Technical Validation

### ECG and ECG annotation quality

The quality assessment for the waveform data was done during the data selection process. As stated previously, the data was first validated by an experienced specialist cardiac nurse and then validated again by an experienced cardiologist. Records presenting a high level of noise were rejected during this phase. All the AF crisis (AF onset and AF termination) were annotated by the cardiologist and reviewed by the specialist cardiac nurse if a second opinion was needed. The labels were then cross-validated during the creation and clean-up of the database, as discussed in the *Methods* section.

### AF detection with ML and DL models for validation

We evaluated the ECG waveform annotations and the RR intervals annotations using ML and DL models. The task given to the model is to detect the presence of AF in an ECG window or RR intervals window. The first model was trained and tested on the RR intervals and RR intervals annotations. We created a gradient boosting tree (XGBoost) model and derived heart rate variability (HRV) features from the RR intervals. The HRV features were extracted from the time domain, frequency domains and the Poincaré plot. We used a 10-fold cross-validation with stratification on the patient level, i.e. all the records from one patient can only be found in either the train or the test split.

The second model is a DL model. We choose to implement a 1-dimensional convolutional neural network (CNN), using an input window of 40 seconds, i.e. 8196 samples. The model is composed of 9 blocks of CNN with two branches, where the second branch is a skip connection to improve the training and results. It was inspired by the model presented by Attia *et al*. for AF identification^6^, which shows impressive performance. The model was trained during 20 epochs with early stopping and optimized using Adam. We used 5 repetitions of boostraping to evaluate the confidence intervals of the metrics. For each one of the model trainings, a new train-validation-test split was created. As for the first ML model, the records were separated at the patient level to avoid any contamination of the test set. The results of the two models are presented in Table 3.

**Table 3:** Comparison of the results for AF detection task using two models: ML model (XGBoost) vs DL model (CNN).

Model	Input	Window size	AUROC	Accuracy	Sensitivity	Specificity	F1 score
XGBoost	RR	300 RR (≈5 minutes)	0.967 (0.950–0.983)	0.972 (0.961–0.983)	0.951 (0.917–0.984)	0.983 (0.975–0.990)	0.957 (0.938–0.975)
CNN	ECG	8192 samples (≈40 seconds)	0.995 (0.990–0.999)	0.982 (0.972–0.992)	0.952 (0.919–0.985)	0.992 (0.988–0.997)	0.971 (0.954–0.989)

The value in parentheses represents the 95% confidence interval. AUROC is the area under the ROC curve.

**Fig. 12:**
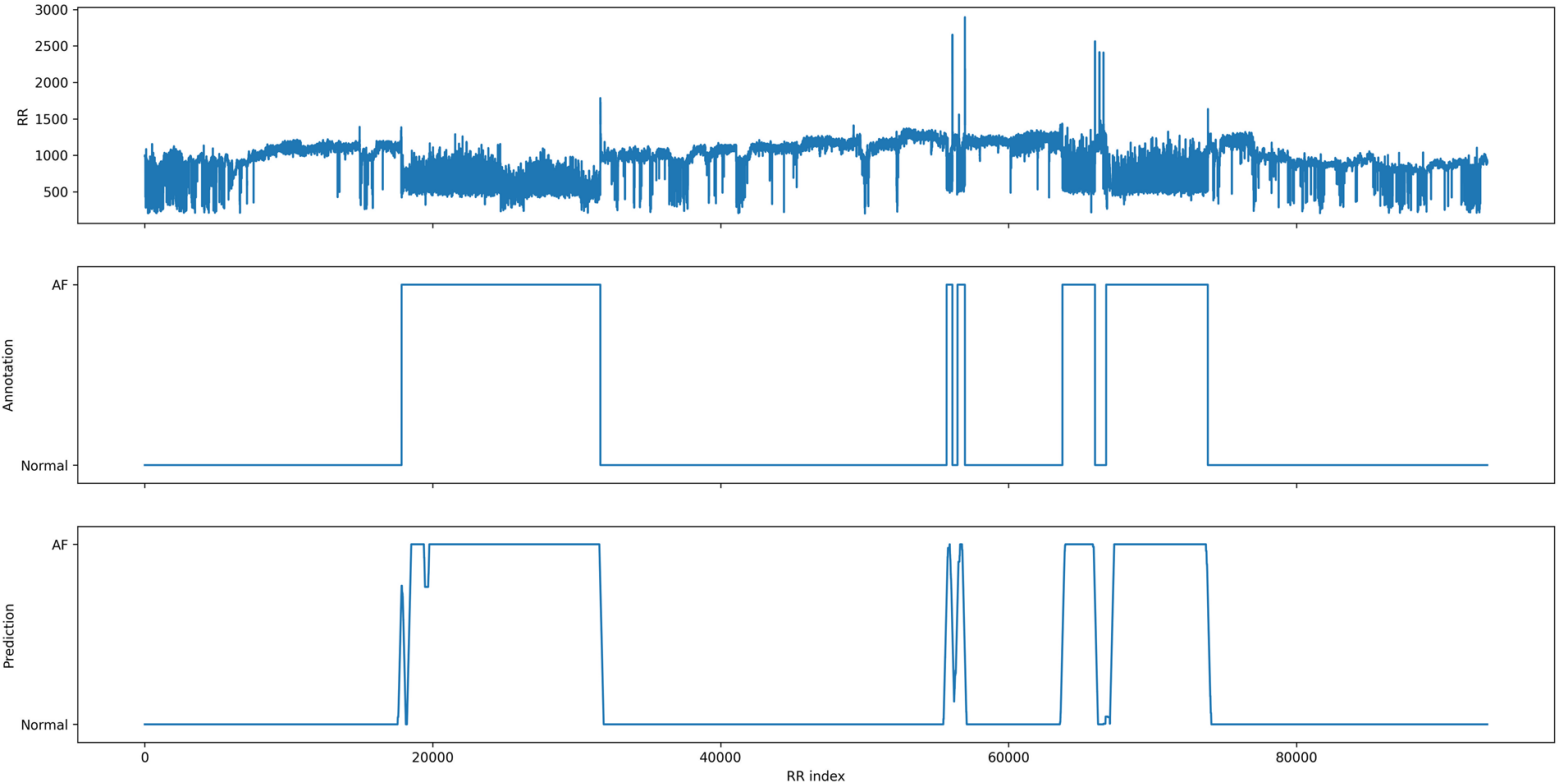
Prediction of the ML model on an unseen record record_104.

**Fig. 13:**
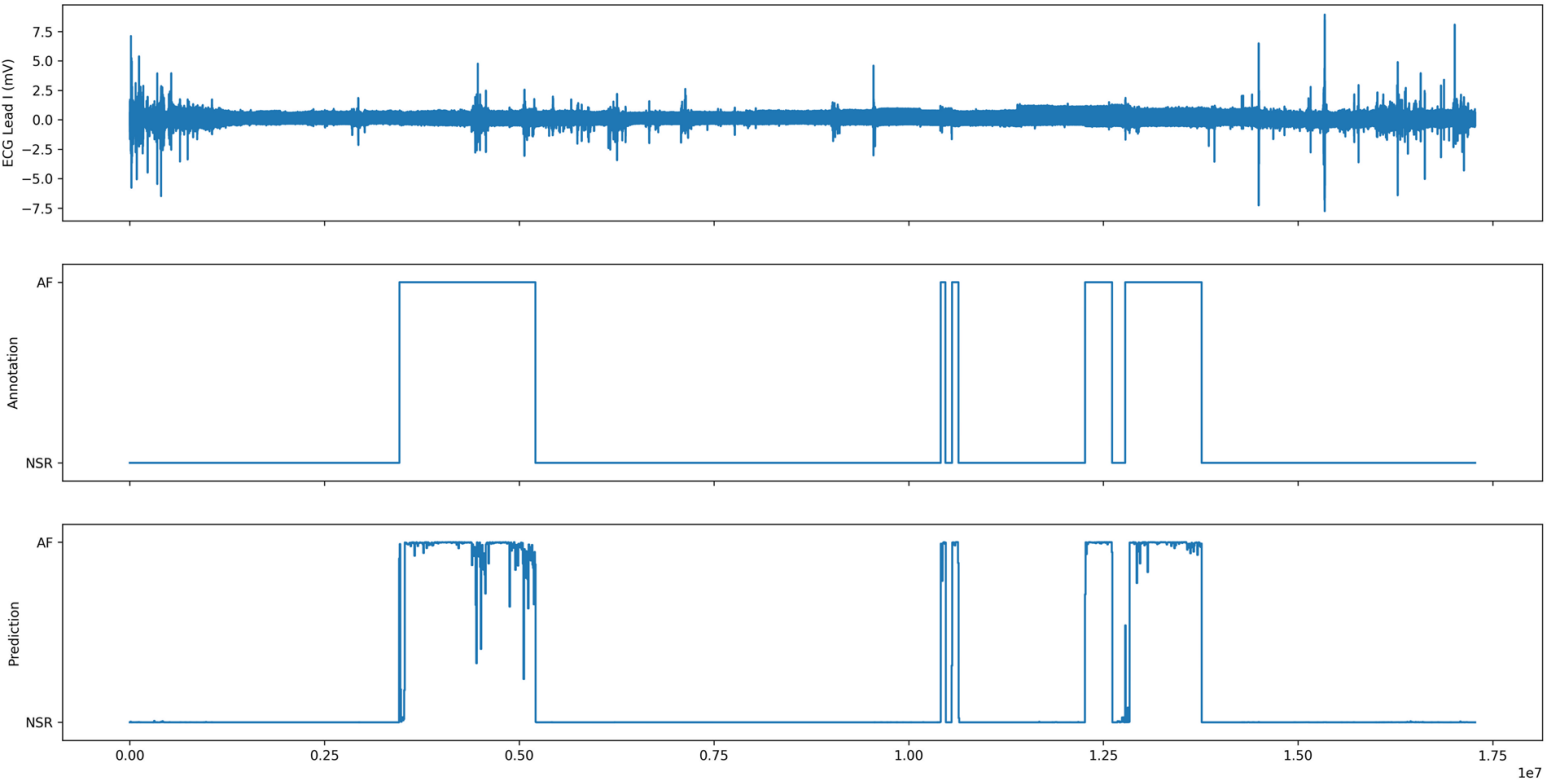
Prediction of the DL model on an unseen record record_104.

Finally, we evaluated both models on an unseen patient record. We used a sliding window to create the annotation of the model on the whole record and compared it visually to the cardiologist annotation. The results for the ML model are presented in Fig. 12 and the results from the DL model are presented in Fig. 13. Both models were able to create new annotation corresponding to the cardiologist annotation with the 5 AF episodes present in the record. It confirms the ability of ML and DL models to be used as a tool for medical decision support.

## Usage Notes

We have developed Python helper functions to facilitate tasks such as reading ECG waveform files along with their corresponding annotations, as well as reading RR files with their corresponding annotations. Visualization functions were also developed to visualize annotations, i.e. AF onset and AF termination and complete records. The two models are presented as an example in the code repository. We suggest the use of Python to carry out analysis of the database, with the use of libraries such as NeuroKit2^15^. In addition, libraries such as scikit-learn^16^, Tensorflow^17^ and Pytorch^18^ can be recommended to build ML and DL models.

## Data Availability

The code described in the usage notes is available on GitHub (https://github.com/cedricgilon/iridia-af). It includes *utils* tool and example code to start using IRIDIA-AF database.
