# Publisher Correction: IRIDIA-AF, a large paroxysmal atrial fibrillation long-term electrocardiogram monitoring database

**DOI:** 10.1038/s41597-023-02772-1

**Published:** 2023-12-04

**Authors:** Cédric Gilon, Jean-Marie Grégoire, Marianne Mathieu, Stéphane Carlier, Hugues Bersini

**Affiliations:** 1 IRIDIA, Université libre de Bruxelles, Brussels, Belgium; 2 Cardiology Department, Université de Mons, Mons, Belgium

**Keywords:** Atrial fibrillation, Databases, Machine learning, Databases, Atrial fibrillation, Databases, Machine learning, Databases

Correction to: *Scientific Data* 10.1038/s41597-023-02621-1, published online 18 October 2023

Fig. 12 and Fig. 13 were incomplete; the figures should have appeared as shown below. The original article has been corrected.

**Fig. 12:**
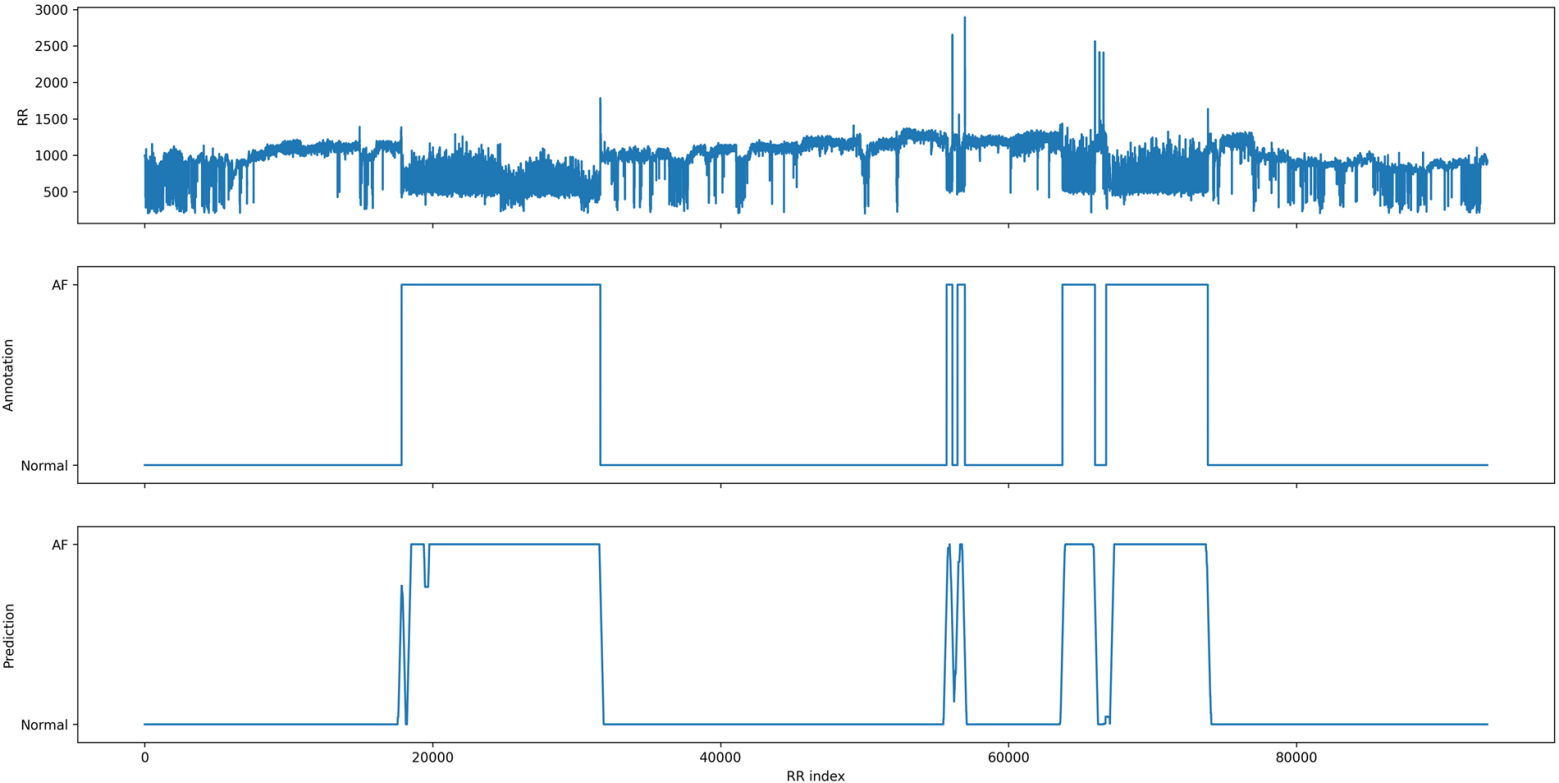
.

**Fig. 13:**
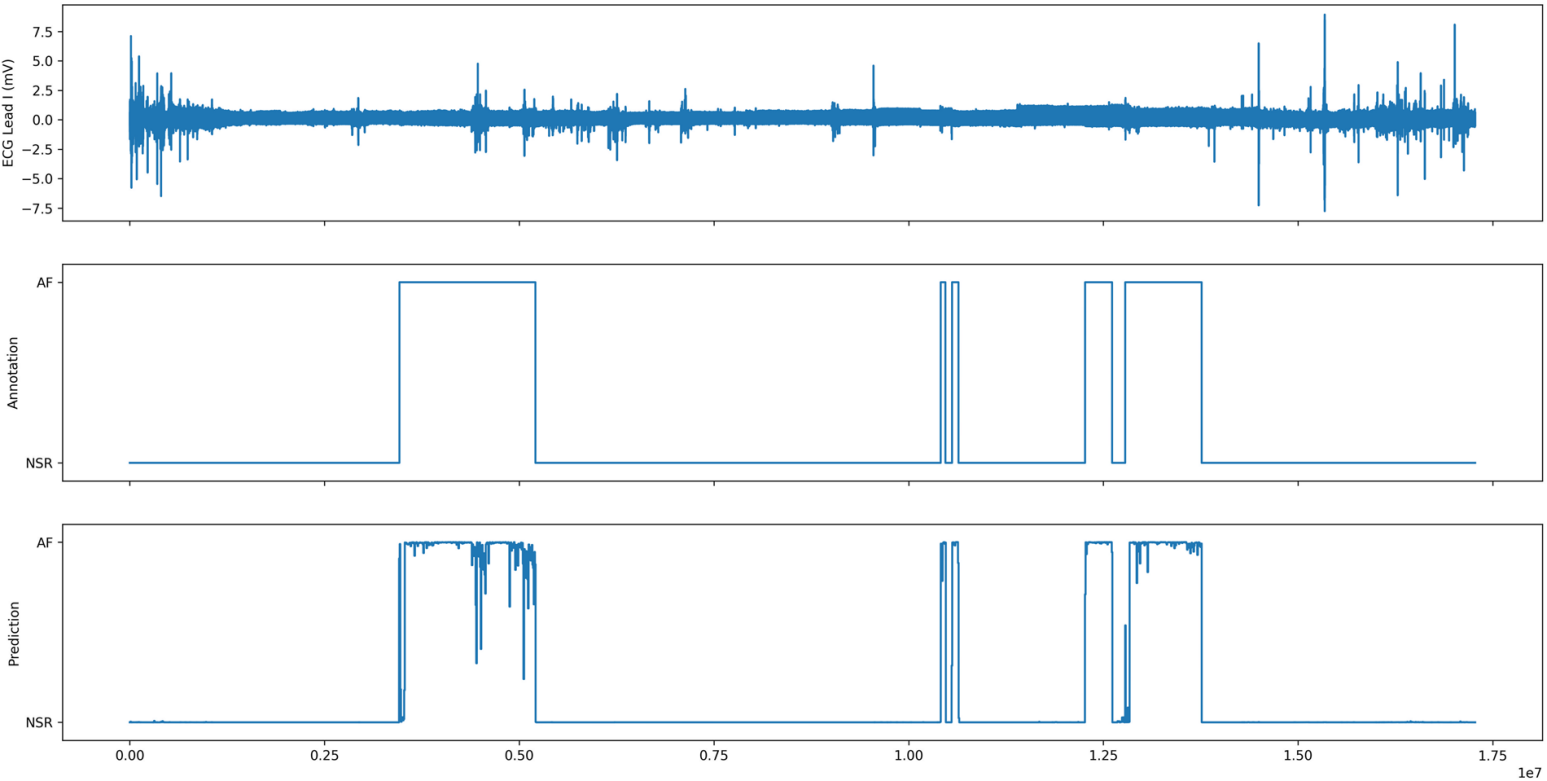
.

